# On the reproductive success of early-generation hatchery fish in the wild

**DOI:** 10.1111/eva.12183

**Published:** 2014-07-09

**Authors:** Mark R Christie, Michael J Ford, Michael S Blouin

**Affiliations:** 1 Department of Integrative Biology, Oregon State UniversityCorvallis, OR, USA; 2 Department of Biological Sciences and Department of Forestry and Natural Resources, Purdue UniversityWest Lafayette, IN, USA; 3 Conservation Biology Division, National Marine Fisheries Service, Northwest Fisheries Science CenterSeattle, WA, USA

**Keywords:** captive breeding, domestication, fitness, hatcheries, relative reproductive success, salmon.

## Abstract

Large numbers of hatchery salmon spawn in wild populations each year. Hatchery fish with multiple generations of hatchery ancestry often have heritably lower reproductive success than wild fish and may reduce the fitness of an entire population. Whether this reduced fitness also occurs for hatchery fish created with local- and predominantly wild-origin parents remains controversial. Here, we review recent studies on the reproductive success of such ‘early-generation’ hatchery fish that spawn in the wild. Combining 51 estimates from six studies on four salmon species, we found that (i) early-generation hatchery fish averaged only half the reproductive success of their wild-origin counterparts when spawning in the wild, (ii) the reduction in reproductive success was more severe for males than for females, and (iii) all species showed reduced fitness due to hatchery rearing. We review commonalities among studies that point to possible mechanisms (e.g., environmental versus genetic effects). Furthermore, we illustrate that sample sizes typical of these studies result in low statistical power to detect fitness differences unless the differences are substantial. This review demonstrates that reduced fitness of early-generation hatchery fish may be a general phenomenon. Future research should focus on determining the causes of those fitness reductions and whether they lead to long-term reductions in the fitness of wild populations.

## Introduction

Substantial numbers of hatchery-reared salmon are intentionally released into the wild each year (Naish et al. 2008; Kostow 2009). For example, over 5 billion hatchery salmon are released annually into the Northern Pacific (Heard 1995; Augerot and Foley 2005). Many of the released fish are intended solely for future harvest, but a growing number of individuals are intended to spawn in the rivers where they were released to bolster declining wild populations (Waples and Drake 2004). Regardless of the future goals associated with the released hatchery fish, the fact remains that a large number of hatchery fish spawn in the wild each year, often with wild fish (Araki et al. 2008; Fraser 2008). Furthermore, it is now well established that hatchery fish often have much lower fitness in the wild when compared to their wild-born counterparts owing to two main causes. First, many populations are locally adapted (Taylor 1991; Fraser et al. 2011; Bourret et al. 2013), and hatchery programs that use nonlocal broodstock (i.e., adults sourced from a different population) can create fish with lower reproductive success in the wild when compared to fish produced by local-origin broodstock (Berejikian and Ford 2004; Araki et al. 2008). Second, it is widely recognized that divergence can rapidly occur between hatchery and wild fish owing to genetic adaptation to captivity (‘domestication’; Fleming and Einum 1997; Lynch and O'Hely 2001; Frankham 2008; Fraser 2008; Christie et al. 2012a). Older hatchery stocks that have experienced multiple generations of selection in a hatchery often have much lower fitness in the wild than more recently established stocks (Berejikian and Ford 2004; Araki et al. 2008).

Heritably low fitness of hatchery fish is a key conservation and management concern. If hatchery programs release fish with heritably lower reproductive success, then those effects may propagate through future generations and reduce the fitness of the entire population (Lynch and O'Hely 2001; Ford 2002; Waples and Drake 2004). Consequently, a growing number of hatchery programs are moving away from using domesticated hatchery stocks in favor of local-origin fish collected directly from the wild. Wild- and local-origin broodstocks are expected to produce hatchery offspring that have fitness values much closer to those of wild fish. Therefore, these ‘early-generation’ hatchery offspring may represent a lower genetic risk to wild populations (Mobrand et al. 2005; Baskett and Waples 2013). The potential benefits of using wild- and local-origin fish in hatcheries are especially relevant for ‘supplementation programs’, which typically have a goal of increasing the abundance of a wild population rather than only producing fish for harvest (Waples and Drake 2004; Waples et al. 2007). Whether early-generation hatchery fish actually have fitness values similar to those of wild fish is difficult to test, remains a point of controversy, and is the subject of this review.

One parameter that is key to evaluating the fitness consequences of hatchery programs on wild populations is relative reproductive success (RRS; Cuenco 1994). RRS is defined as the reproductive success of hatchery-origin fish (i.e., fish whose parents spawned in a hatchery, hereafter ‘hatchery’) relative to wild-origin fish (i.e., fish whose parents spawned in the wild, hereafter ‘wild’) when both groups are allowed to spawn in the wild (Box 1). For example, if RRS were equal to 0.9, then a hatchery fish spawning in the wild would produce an average of 9 offspring for every 10 offspring produced by a wild fish. In practice, estimating the RRS of hatchery and wild fish (see Box 1) can be challenging because it requires (i) a genetic sample (e.g., tissue samples) from most of the reproductive adults in the population (i.e., F1 fish), (ii) representative samples of the offspring produced by the adults (i.e., F2 fish), and (iii) accurate assignment of offspring back to their parents. The first two challenges represent logistical difficulties, and studies have mostly relied on dams or other substantial barriers to facilitate sampling efforts. Difficulties associated with parentage analysis in large, natural populations (reviewed in Jones and Ardren 2003; Jones et al. 2010) can also present a challenge to accurately estimating RRS (Araki and Blouin 2005; Christie et al. 2013; Steele et al. 2013).

**Box 1: Study designs for estimating relative reproductive success**

Relative reproductive success studies are, by design, multigenerational in nature, but it can be challenging to know precisely which generation is being referred to because different studies use different nomenclatures. We recommend adopting a common nomenclature to facilitate comparisons across studies. Here, we suggest that the first generation of hatchery fish to spawn in the wild be referred to as ‘F1 hatchery fish’ and their offspring as the ‘F2’ generation. The illustration in this box shows the progression and design of RRS studies. For supplementation programs, broodstock fish are typically collected as adults in the wild and brought into a hatchery, where they are spawned. In some programs, not enough wild fish are available and so returning F1 hatchery fish are used in addition to wild fish as broodstock. The offspring of these broodstock, F1 hatchery fish, are typically reared for up to a year in the hatchery before being released at or near the adult spawning grounds in the wild. After release, the hatchery fish migrate to the ocean, along with their wild counterparts, where they forage and grow before ultimately returning to freshwater to spawn. Upon returning as adults, the F1 hatchery fish spawn in the wild alongside (and often with) wild fish. Both the F1 hatchery and wild fish produce F2 wild fish, which spend their entire lives in the wild. F2 fish can be sampled at a variety of developmental stages. Using genetic parentage analyses, F2 wild fish can be assigned back to their F1 parents and the reproductive success of the F1 hatchery and wild fish can be compared. RRS calculations are typically performed for each sex separately because the sexes may respond differently to the hatchery environment.

There are also some variations in the typical study design that have been employed. For example, Araki et al. (2009) examined the reproductive success of F2 fish in the wild (extending the figure below by an additional generation). This study design demonstrated that the fitness of wild-born fish depends on whether their parents were hatchery or wild (i.e., there are genetic ‘carryover’ effects; see ‘Environmental versus genetic effects’ for details). Another way to examine reproductive success data is to compare the cross types for pairs of F1 fish spawning in the wild (i.e., H × H, H × W, and W × W; e.g., Araki et al. 2007a). However, this approach has a serious drawback in that many pairs may produce 0 surviving offspring. In most salmon populations, distributions of family sizes are highly skewed (well approximated by a negative binomial; see Figure S1). Therefore, the vast majority of fish leave zero surviving offspring. When performing RRS analyses separately for each sex (see paragraph above), this is not an issue because an F1 adult will simply have 0 F2 fish assigned back to itself. However, with pair data, this becomes a serious issue because pairs can only be identified using parentage analysis if they produced at least one surviving offspring. One cannot distinguish between pairs that mated and produced 0 surviving offspring and ‘pairs’ that never mated in the first place. There are two consequences of this phenomenon. First, RRS estimates from pair data will be biased if one cross type leaves 0 offspring at a higher rate than the other cross type (i.e., RRS is upwardly biased toward the fish type having a greater proportion of 0 offspring; see Figure S4 for an example). Second, pair data tend to have very small sample sizes, thus compounding the problem of imprecise parameter estimates and low statistical power for hypothesis testing that plague RRS studies (see Box 2).

Despite these drawbacks, several authors have included pair data in RRS studies. Araki et al. (2007a) compared the fitness of hatchery females that mated with hatchery males *versus* hatchery females that mated with wild males. They repeated the exercise using wild females that mated with each type of male. Hatchery males left fewer offspring than wild males only when mating with hatchery females, suggesting a possible negative interaction for fitness, but the result was not statistically significant. Araki et al. (2007b) performed similar analyses, but the sample sizes were so small that few useful comparisons could be made. However, when examining the offspring produced by each type of pairing, there was an apparent excess of offspring from matings between two wild fish (here, they used the overall RRS estimates from the single-sex data to generate the expected numbers per cross type in the absence of nonadditive fitness effects). Hess et al. (2012) also examined the reproductive success for different pairings and observed no significant difference in fitness between cross types. However, in all cases, the sample sizes were small (see Box 2). Thus, examining pair data may be informative if the effect size is very large (i.e., different pairings have vastly different outcomes for reproductive success) *and* if one is willing to make the assumption that the number of failed matings occurs equally between fish types. In general, the challenges presented by the small sample sizes, low power, and inaccurate estimates suggest that analyses of the different F1 pairings should be more exploratory than hypothesis testing in nature.

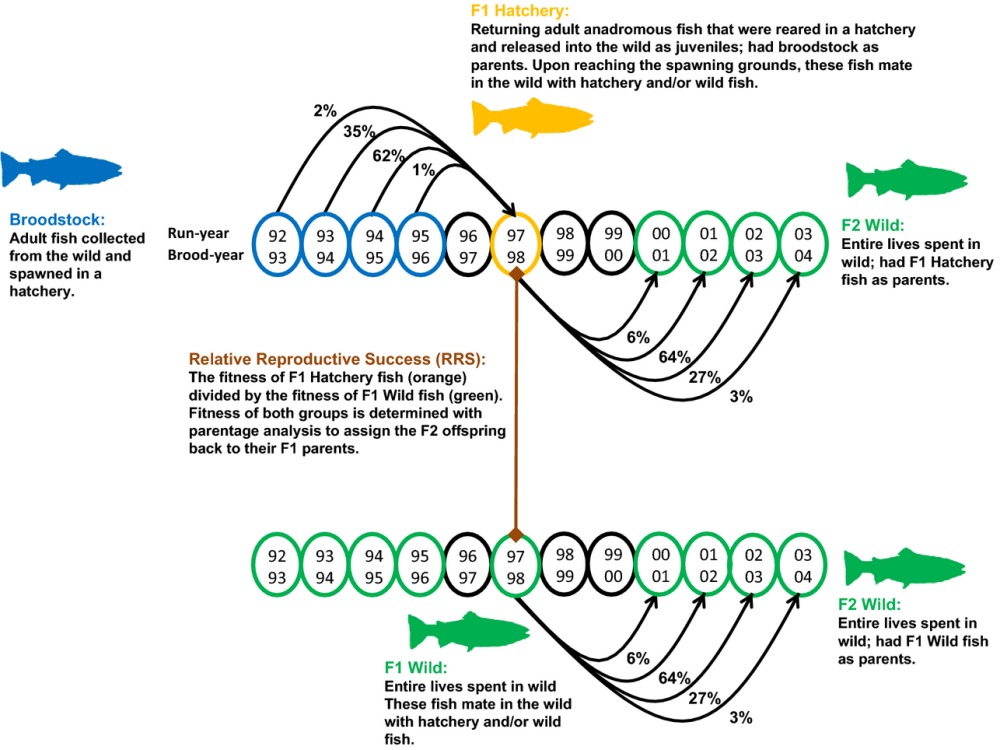

Illustration of how the relative reproductive success (RRS) of first-generation hatchery relative to wild fish is calculated. Arrows illustrate the overlapping generations that occur in most salmon species. For any given run year, adults were born in multiple years and their offspring return to spawn in different years. The percentages in the illustration show the fraction of each cohort that return in each year (based on steelhead from the Hood River, Oregon, as an example). RRS is calculated by dividing the average number of offspring per hatchery fish by the average number of offspring per wild fish in a given run year.

Despite these challenges, we identified 51 RRS point estimates from six studies and four species to answer the following questions: (1) What is the average RRS for hatchery salmon originating from local- and predominantly wild-origin broodstock? (2) Do RRS estimates vary substantially across studies or species? (3) How do sample sizes affect the ability to detect differences in reproductive success between hatchery and wild fish? (4) Do RRS estimates differ between males and females? (5) What are the relative contributions of genetic and environmental effects to reduced fitness? and (6) What stage in the life cycle do fitness-related effects occur? We conclude with a discussion of other relevant studies (e.g., those conducted in spawning channels), review additional insights into the mechanism responsible for the reductions in fitness observed in hatchery fish, and provide recommendations for future RRS studies.

## Case studies

We identified six studies that directly calculated RRS for fish spawning in the wild from programs that used local-origin broodstock. Although we focused on systems where broodstock were collected from the wild, the precise amount of hatchery ancestry in the broodstock varied. Some hatcheries used wild fish exclusively, while others could not always discern between wild and hatchery fish or had small population sizes such that some returning hatchery fish were incorporated into the broodstock. For each case study, we report the average proportion of wild fish used in the breeding program. A brief overview of each case study is provided in Table 1, and detailed background information including the sampling, hatchery, and life-history information for each population is provided as Supporting Information. To facilitate comparisons across studies, we also calculated a weighted geometric mean and maximum-likelihood estimate of RRS for each study (see ‘RRS estimates across years and studies’).

**Table 1:** Details for each of the six case studies. We report the number of F1 run years evaluated (years), the life stage at which F2 fish were collected, the hypothesis testing methods employed, and any features unique to the study. Four species from six populations are represented

Case	Common name	Species	Years	F2 life stage	Hypothesis testing	Unique features	References
1	Chinook	*O. tshawytscha*	3	Adult, juvenile	*t*-tests, linear models, GLM	Identified spawning location	Ford et al. (2013)
2	Coho	*O. kisutch*	3	Adult	Randomization tests, anova	Unfed fry, different broodstock crosses	Thériault et al. (2011)
3	Steelhead	*O. mykiss*	6	Adult	Randomization tests	Different broodstock crosses	Araki et al. (2007a,b)
4	Atlantic salmon	*S. salar*	3	Juvenile	Bootstrapping, GLM	Only Atlantic Ocean study to date	Milot et al. (2013)
5	Steelhead	*O. mykiss*	6	Adult, juvenile	GLM	Integrated broodstock program	Berntson et al. (2011)
6	Chinook	*O. tshawytscha*	4	Adult	Randomization tests	No prior hatchery intervention	Hess et al. (2012)

### Case 1: Wenatchee River, Chinook salmon

#### Study design

The first study examines the reproductive success of Chinook salmon (*Oncorhynchus tshawytscha*) from the Wenatchee River in Washington State (Williamson et al. 2010). A combination of wild × wild, wild × hatchery, and hatchery × hatchery crosses was performed in the hatchery, which resulted in an average of 30% wild fish contributing as broodstock. Because the amount of hatchery ancestry in the broodstock did not have a detectable effect on the reproductive success of F1 fish (Ford et al. 2012), all F1 hatchery fish were analyzed together (see ‘Environmental versus genetic effects’ for separate analyses). Reproductive success for individual fish was highly variable*,* a result that is consistent with other pedigreed salmon populations (e.g., Seamons et al. 2004; McLean et al. 2008; Christie et al. 2012b). The F1 sample sizes ranged from 485 to 1914 individuals.

#### RRS estimates

**Figure 1:**
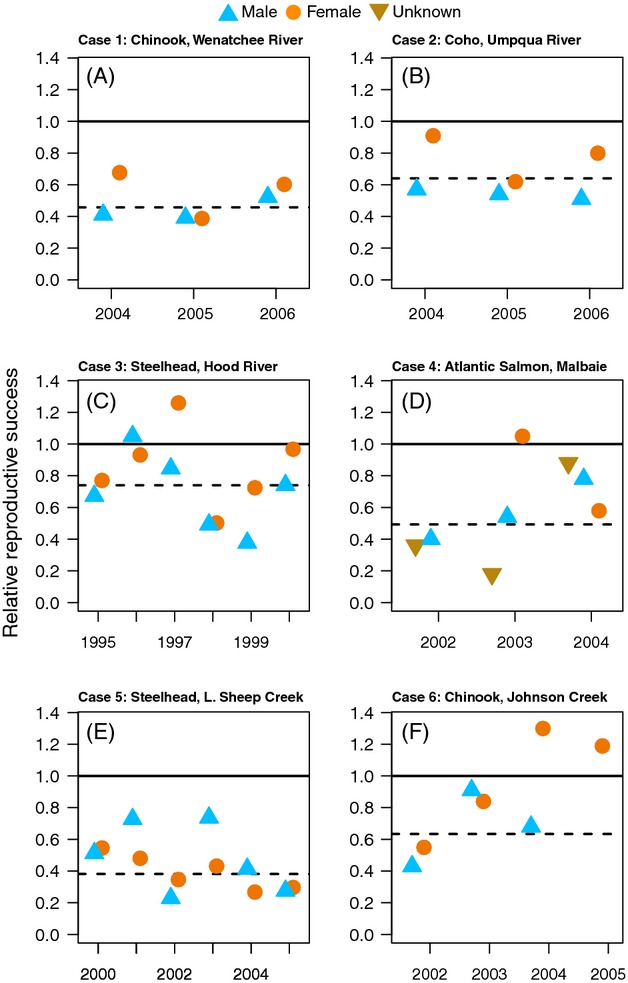
The relative reproductive success (RRS) values for the six case studies. These studies directly measured the reproductive success of F1 hatchery fish relative to wild fish. If F1 hatchery and wild fish had equal fitness, then RRS would be equal to 1 (solid lines). Here, orange circles represent females, blue triangles represent males, and tan triangles represent undetermined sex. For each study, we calculated a geometric mean, weighted by F1 sample size (dashed lines).

Relative reproductive success for hatchery males and females ranged from 0.39 to 0.52 and 0.38 to 0.67, respectively, for the 2004–2006 spawning years (Fig. 1A; Ford et al. 2013). The weighted geometric mean RRS for all years equaled 0.45.

#### Additional information

All six point estimates were significantly lower than 1. Hatchery fish continued to have significantly lower fitness after accounting for weight, run-timing, and age (three variables that also had an effect on fitness). Particularly for males, hatchery fish tended to return to spawn at younger ages, a factor that partly explained their lower reproductive success. This study also found that spawning location was correlated with reproductive success; fish that spawned upstream tended to have higher reproductive success than fish that spawned downstream. A greater proportion of wild fish spawned upstream and a part of the low RRS of hatchery fish in this population are explained by this difference in spawning location.

### Case 2: Umpqua River, coho salmon

#### Study design

The second study examined the RRS of coho salmon (*Oncorhynchus kisutch*) from the Umpqua River in Oregon (Thériault et al. 2010, 2011). Hatchery fish were released as both smolts and unfed fry (see Supporting Information for details). Half of the broodstock crosses were wild × wild, and the other half were hatchery × hatchery, resulting in an average of 50% wild contribution to broodstock. Because there was no effect of the type of broodstock cross performed, all F1 hatchery fish were pooled together (see ‘Environmental versus genetic effects’ for separate analyses). The F1 sample sizes ranged from 455 to 713 individuals.

#### RRS estimates

Relative reproductive success for hatchery males and females ranged from 0.51 to 0.57 and 0.62 to 0.91, respectively, for the 2004–2006 spawning years (Fig. 1B). The weighted geometric mean RRS for all years equaled 0.64.

#### Additional information

Over all years and fish-rearing strategies, both female and male hatchery fish had significantly lower reproductive success than their wild-born counterparts (females *P* < 0.0001; males: *P* < 0.001). Although the unfed fry had slightly higher fitness than those released as smolts, they still had lower fitness than wild fish (females: RRS = 0.84, *P* < 0.26; males: RRS = 0.62, *P* < 0.001).

The RRS for hatchery jacks (fish that spent only a single year at sea) ranged from 0.72 to 2.13 when compared to wild jacks and was not significantly higher or lower than one. However, the number of returning jacks was roughly one-third of the number of returning 3-year-old males, and thus, both the statistical power to detect differences and the precision of the point estimates may have been low (see Box 2).

### Case 3: Hood River, steelhead

#### Study design

The relative fitness of winter-run steelhead (anadromous *Oncorhynchus mykiss*) has been extensively studied in the Hood River, Oregon. Here, we present the results from F1 hatchery fish produced only by crosses between two wild fish because there were significant differences in the reproductive success of F1 hatchery fish created with wild or hatchery broodstock (see ‘Environmental versus genetic effects’ for separate analyses). The F1 sample sizes ranged from 120 to 868 individuals.

#### RRS estimates

Relative reproductive success for hatchery males and females ranged from 0.37 to 1.05 and 0.50 to 1.26, respectively, for the 1995–2000 spawning years (Fig. 1C). The weighted geometric mean RRS for all years equaled 0.74.

#### Additional information

The first fitness-based study from the Hood River examined only the first 3 years of the supplementation program, 1995–1997 (Araki et al. 2007a). Corrections for angling were made for these first 3 years that were likely too conservative because they assumed that all of the fishing pressure was directed toward the hatchery fish. With the corrections for angling, the RRS estimates were close to 1. However, even without the corrections for angling, the overall RRS for 1995–1997 was fairly close to 1 for both males and females (0.865 and 0.984, respectively, Fig. 1C). RRS estimates for an additional three run years of data were reported for 1998–2000 (Araki et al. 2007b). In these years, there was no angling, and RRS values were lower, ranging from 0.492 to 0.968 (Fig. 1C). The study-wide weighted geometric mean that included the correction for angling equaled 0.85 (Araki et al. 2007b) such that the true study-wide estimate likely falls somewhere between 0.74 and 0.85.

### Case 4: Malbaie River, Atlantic salmon

#### Study design

The fourth study examined the RRS of Atlantic salmon (*Salmo salar*) from the Malbaie River, which is located on the northern shore of the St. Lawrence estuary in Quebec, Canada (Milot et al. 2013). All broodstock fish were collected directly from the wild, but there was a possibility that a small portion of adults collected were themselves first-generation hatchery fish that had been released as unmarked fry or smolts. Not all F1 fish could be confidently assigned to a sex, resulting in an additional ‘unknown sex’ category. The F1 sample sizes ranged from 135 to 348 individuals.

#### RRS estimates

Relative reproductive success for hatchery males and females ranged from 0.40 to 0.78 and 0.58 to 1.05, respectively, for the 2002–2004 spawning years (Fig. 1D). The RRS values for fish of unknown sex ranged from 0.18 to 0.88. The weighted geometric mean RRS for all years equaled 0.49.

#### Additional information

The sex ratio of Atlantic salmon typically is skewed in favor of males (Fleming 1998), and this study was no exception, reporting 75% of identified adults to be male. Additionally, F1 hatchery fish returned in greater numbers after a single winter at sea compared with their wild counterparts. Because fish that only spent a single winter at sea had lower reproductive success than those that spent longer durations at sea, differential age at maturation explains at least some of the difference in reproductive success between wild and hatchery individuals. Similar to Thériault et al. (2011; case study 2), hatchery fish released at an earlier stage (here as hatchery-stocked fry) had higher reproductive success relative to wild fish than did hatchery fish released as smolts, although RRS values for both groups were still less than one.

### Case 5: Little Sheep Creek, steelhead

#### Study design

The next study examined the lifetime reproductive success in steelhead, from Little Sheep Creek in Oregon (Berntson et al. 2011). The hatchery stock was created using local-origin, wild fish. However, because of low numbers of returning wild fish each year, this system used an average of 10% wild fish and 90% hatchery fish as broodstock over the years for which RRS was calculated. The hatchery fish in this program had the highest amount of hatchery ancestry in all of the six case studies. Consequently, a large portion of ‘wild’ fish may have had considerable hatchery ancestry. The F1 sample sizes ranged from 84 to 857 individuals.

#### RRS estimates

Relative reproductive success for hatchery males and females ranged from 0.23 to 0.74 and 0.27 to 0.54, respectively, for the 2000–2005 spawning years (Fig. 1E). The weighted geometric mean RRS for all years equaled 0.38.

#### Additional information

Relative reproductive success was measured for 10 years for adult-to-juvenile fitness estimates and for 6 years for adult-to-adult estimates. There were no detectable differences in RRS between male and female fish. Overall, the RRS estimates in each year were relatively consistent, and no single-year point estimate was greater than 0.80.

### Case 6: Johnson Creek, Chinook salmon

#### Study design

The last case study we included examined the RRS of Chinook salmon from Johnson Creek, Idaho (Hess et al. 2012). Nearly all broodstock crosses were performed with wild fish (i.e., only 7 hatchery fish were incorporated into the broodstock program), resulting in 98% wild broodstock. RRS was measured for 3 years for males and for 4 years for females. All F2 offspring were sampled as adults. The RRS values reported in the main text of Hess et al. (2012) were calculated using only the F1 parents that produced at least one returning offspring. However, this approach was not taken in any of the other case studies, and for the purposes of comparison, we report the RRS values that use all of the adult fish (reported in the supplementary material of Hess et al. 2012). The F1 sample sizes ranged from 12 to 410 individuals.

#### RRS estimates

Relative reproductive success for hatchery males and females ranged from 0.43 to 0.91 and 0.55 to 1.30, respectively, for the 2002–2005 spawning years (Fig. 1F). The weighted geometric mean RRS for all years equaled 0.634.

#### Additional information

This study was unique in that nearly all of the broodstock fish were wild and this system did not have a prior history of hatchery ancestry. Nevertheless, there are still substantial fitness-related effects associated with hatchery rearing, further suggesting that genetic or environmental effects can occur in a single generation (see also ‘Environmental versus genetic effects’ below). The fitness effects appear to be more severe for males than for females. In one run year, 2003, data were available to calculate RRS values for jacks (3-year-old fish). The RRS value for jacks was equal to 0.32, and there were nearly 4 times as many returning hatchery jacks as there were wild jacks.

## RRS estimates across years and studies

To combine RRS estimates across years, one must decide how to combine the estimates and whether or not to weight them by F1 or F2 sample sizes (see Box 1 for descriptions of F1 versus F2). There are two general approaches for combining estimates: (i) calculate an appropriately weighted geometric mean of the RRS point estimates or (ii) use the maximum-likelihood method of obtaining an RRS estimate across years (Kalinowski and Taper 2005), an approach that also facilitates the construction of confidence intervals. Calculating the geometric mean, as opposed to an arithmetic mean, is appropriate because it eliminates bias caused by the arbitrary placement of hatchery fitness in the numerator when estimating RRS (see Table S1 for a detailed example). Weighting by F1 sample sizes is sensible under an assumption that a given population has an underlying ‘true’ RRS and that the true RRS can be obfuscated by the among-individual variance in reproductive success. For example, an RRS estimate based on a sample of only 10 F1 fish could vary greatly across multiple independent trials given the stochastic survival of offspring (Box 2). With this point in mind, it makes sense to weight estimates by F1 sample sizes when combining RRS estimates across years because estimates based on small F1 sample sizes will be less precise and should therefore contribute less to the overall estimate. An alternative point of view is to evaluate only the RRS of a specified set of fish in a particular year or set of years. By focusing on the F2 sample sizes, one can accurately estimate the realized RRS of a particular group of fish in a particular set of years. This latter approach is the one taken by Kalinowski and Taper (2005), in their method of calculating maximum-likelihood confidence intervals for RRS estimates. In particular, their method assumes the frequencies of F1 hatchery and wild fish are known exactly (as will be the case if the entire population is sampled at a dam or weir) and that the only uncertainty in the estimate of RRS comes from sampling a finite number of offspring. Under this set of assumptions, if the number of F2 offspring sampled is large, precise estimates of the realized RRS for a particular set of F1 fish can be obtained, regardless of F1 sample size.

**Box 2: Statistical power and RRS**

**Statistical power to detect a difference in RRS**

Statistical testing in RRS studies uses the null hypothesis that the RRS of the groups being compared is equal. If the test statistic exceeds a specified critical value, the researcher concludes that the null hypothesis is false and that there really is a difference in fitness between wild and hatchery fish. If the test statistic does exceed the critical value, the researcher cannot reject the null hypothesis. It is essential to note, however, that being unable to reject the null hypothesis does not mean that there may not be real differences in fitness between hatchery and wild fish (Sokhal and Rohlf 1995; Ryman et al.^2006^; Kitada et al. 2011; Sham and Purcell 2014). Lack of significance does not necessarily mean lack of effect. It may simply be a matter of low statistical power to detect an effect. To illustrate these issues, we present a heuristic approach in which we controlled true differences in fitness while varying sample sizes. We varied RRS from 0.50 to 0.95 and the sample sizes of F1 fish from 5 to 400 with equal numbers of hatchery and wild F1 fish. We used a negative binomial distribution to determine the number of F2 offspring assigned to each F1 fish (including 0; see histogram in top-right corner of panel B in this box). We chose a negative binomial distribution because it closely mimics the actual distribution of reproductive success values for salmon breeding in the wild (see Figure S1 for comparisons to empirical distributions). To test for differences in mean fitness, we used a *t*-test, although nearly identical results were obtained with a randomization test. For each combination of sample size and RRS, we assigned offspring to ‘hatchery’ and ‘wild’ fish and then used the *t*-test to determine whether the mean number of offspring per hatchery fish was different than the mean per wild fish. This process was repeated 5000 times, and power was calculated as the proportion of trials that had a *P*-value ≤ 0.05. In this context, power represents the probability of correctly identifying a true difference in fitness (i.e., correctly rejecting the null hypothesis when it is in fact false). For the sake of brevity, we do not address the issue of incorrectly rejecting the null hypothesis when it is in fact true.

As illustrated in panel A, the ability to detect a true difference in fitness between hatchery and wild fish depends on (i) the actual difference in fitness between the two groups (here represented by RRS) and (ii) the sample sizes used in the analyses. If RRS is close to one (i.e., the differences in fitness are small), it will not be possible to detect a difference in fitness, unless the sample sizes are very large. Alternatively, if the sample sizes are small, then the true difference in fitness can be substantial, but the limited statistical power may result in a test that is unable to reject the null hypothesis of no difference. For example, panel A shows that even sample sizes greater than 400 had minimal power to detect a 10% difference in fitness (RRS = 0.90) and sample sizes less than 100 were likely to detect only the largest fitness differences (30–50%). To look at it another way, all of the RRS values in this example represented true underlying differences in reproductive success, but only 56% of tests resulted in a ‘significant’ result (*α* ≤ 0.05) and most of those occurred when RRS was 0.70 or less. We use this example to illustrate that a lack of power to detect differences in fitness—because of insufficient sample sizes—cannot be taken to mean that there are no differences in fitness between hatchery and wild fish. How large a fitness difference is biologically important and thus worthy of detection remains unknown. Nevertheless, hatchery fish are often released for many years such that even small effects are expected to accumulate across generations (Lynch and O'Hely 2001; Baskett and Waples 2013). The only empirical data to date come from the Hood River in which F1 fish showing only 15–25% reduced fitness (see case study 3) caused even greater effects on the fitness of the subsequent (F2) generation in the wild (Araki et al. 2009). Thus, even 5–10% differences in fitness could have important cumulative effects over time.

**Precision of RRS point estimates**

Another facet of the same issue facing reproductive success studies is the variability in the point estimates of RRS associated with small sample sizes. When the number of F1 parents is small and there is substantial variance in reproductive success among individuals, the precision for single-year point estimates of RRS will be low. By chance alone, a few hatchery or wild individuals could have high reproductive success in a particular year, having a substantial effect on that year's estimate of RRS. To illustrate this point, we simulated a population with RRS equal to 0.8, although the results were qualitatively similar, regardless of the RRS value chosen. After using the same procedure as described above to assign simulated F2 offspring to each F1 parent, we calculated the estimate of RRS. This process was repeated 10 000 times to obtain the mean and range for 95% of the RRS values. F1 run sizes were varied from 10 to 400 individuals. As the sample size of F1 parents increases, the precision associated with the RRS estimate also increases (panel B). Notice that if an F1 run year has only 50 returning fish, then the range for 95% of the point estimates for RRS varied between 0.5 and 1.3 due to random variation in reproduction among individuals, even though the underlying RRS was in fact 0.8. Thus, RRS estimates based on small numbers of F1 individuals should be interpreted with caution, particularly if the variance in reproductive success among individuals is high.

Half of the point estimates from the highlighted case studies were calculated from fewer than 250 F1 adults (study-wide median**)**, a consequence of both the fact that supplementation programs usually target declining populations and the logistical difficulties associated with sampling large populations in their entirety. Therefore, researchers should consider that single estimates from RRS studies typically lack precision and have low power to detect a difference between wild and hatchery fish. Given the results of these analyses, researchers should (i) be cautious when interpreting ‘nonsignificant’ results from RRS studies, (ii) consider performing *a priori* power analyses before beginning an RRS study, and (iii) report confidence intervals of the RRS estimates to illustrate the range of RRS values consistent with the data.

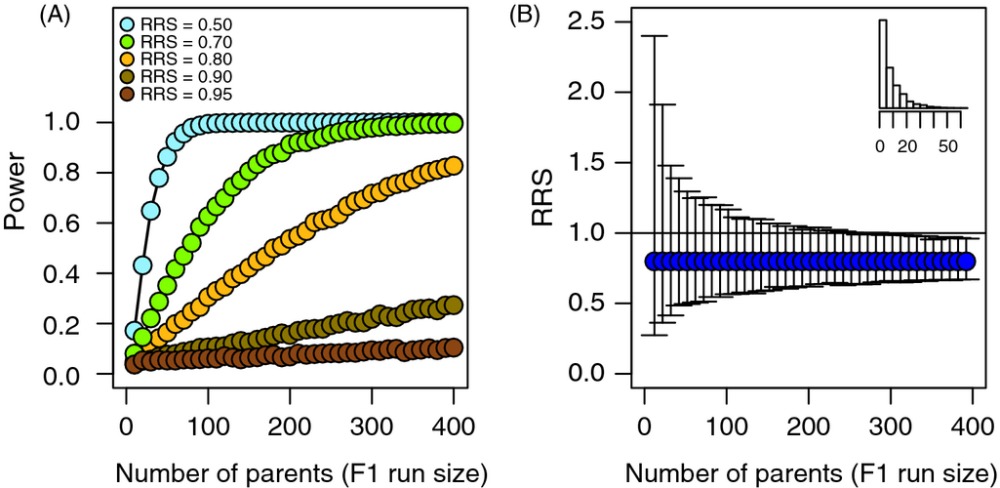

**Figure 2:**
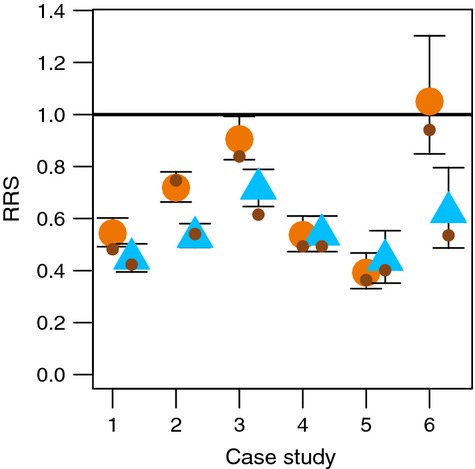
Maximum-likelihood estimates of RRS and their associated 95% confidence intervals combined across all years of each study. Females are represented with orange circles and males as blue triangles. Small brown circles represent geometric means weighted by F1 sample size. Case studies are as follows: 1 Wenatchee River; 2 Umpqua River; 3 Hood River; 4 Malbaie River; 5 Little Sheep Creek; and 6 Johnson Creek (see Table 1 for details).

To investigate these alternative approaches, we present results weighted by both the F1 and F2 sample sizes. We first calculated geometric means weighted by F1 sample sizes for each sex in each case study. Next, we calculated the 95% maximum-likelihood confidence limits using the method of Kalinowski and Taper (2005) for each of the annual RRS estimates from the above case studies (Table S2). We also estimated a single RRS value and associated confidence limit in each study across all years using Kalinowski and Taper's (2005) method. When combining estimates within this framework, it is necessary to assume that the true RRS is constant over all of the years included in the estimate (see eqn. 6 of Kalinowski and Taper 2005; and also Hinrichsen 2003), and this assumption may not be biologically realistic in all cases. The range of the 95% confidence intervals was substantially smaller for the combined multiyear estimates than for the individual single-year estimates (Figure S2). The likelihood-based confidence intervals were calculated separately for each sex, and only one of the 12 estimates had an upper confidence interval that was greater than one (Fig. 2). The confidence intervals also show that estimates determined to be ‘nonsignificant’ are associated with wide confidence intervals and hence low power (Table S2; Box 2). When looking at the individual point estimates of RRS by year, even in cases where the point estimate is greater than 1, the 95% confidence interval usually extends well below 1 (Table S2).

The maximum-likelihood values, which are more influenced by the F2 sample sizes, were very similar to those obtained by geometric means weighted by F1 sample sizes (Fig. 2). Thus, for these studies, the particular weighting scheme does not substantially influence the overall results. Unweighted geometric means also yielded similar results (data not shown). The observation that all approaches to analyzing these data yielded similar results bolsters the conclusion that the reproductive success of early-generation hatchery fish is substantially lower than that of wild fish.

## Effects of hatchery rearing on males versus females

**Figure 3:**
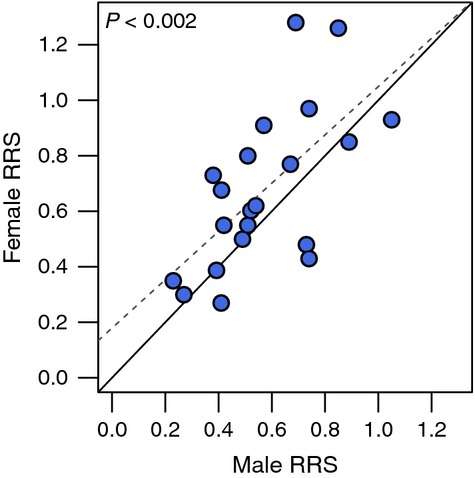
Differences in patterns of relative reproductive success (RRS) between males and females, where RRS represents the reproductive success of hatchery fish relative to wild-origin fish. Each point represents a single run year from each of the case studies. The solid line is the 1:1 line, and 15 of 21 (71%) possible comparisons revealed that females had higher RRS than males from the same run year.

The RRS of male and female hatchery fish compared with their wild counterparts is positively correlated when RRS values for males and females from the same population and run year are compared (*R*^2^ = 0.41, *P* < 0.002; Fig. 3). This correlation suggests that there are environmental or genetic effects associated with hatchery propagation that influences both sexes. Overall, hatchery males tend to have lower RRS values compared with wild males than do hatchery females compared with wild females (Fig. 3). Male hatchery fish RRS is lower than female hatchery fish RRS in 15 of 21 possible comparisons (71%; *P* < 0.046, G test for goodness of fit). Notice that here we are comparing the fitness of hatchery males relative to wild males versus the fitness of hatchery females relative to wild females. This result suggests that males may be more susceptible to environmental or genetic changes caused by hatchery propagation, perhaps due to relaxation of sexual selection (Thériault et al. 2011) or hatchery environments that promote early male maturity (Ford et al. 2012).

## Environmental versus genetic effects

In studies that compare the reproductive success of F1 hatchery and wild fish in the wild (results summarized in Figs 1 and 2), one cannot typically disentangle genetic from environmental effects because the two types of fish experienced different juvenile environments (i.e., genetic and environmental effects are confounded). Furthermore, environmental and genetic effects are not mutually exclusive and both may contribute to the reduced RRS of hatchery fish. Disentangling the relative contribution of genetic and environmental effects to the reduced fitness of early-generation hatchery fish is important for predicting the long-term effects of hatchery fish spawning in wild populations.

Three of the case studies (studies 1, 2, and 3) examined the RRS of hatchery fish with different degrees of hatchery ancestry. These hatchery fish were reared and released into the same environment such that the effect of hatchery ancestry could be isolated (Araki et al. 2007b, 2009; Christie et al. 2012a; Ford et al. 2012). One study (Hood River) estimated RRS after an additional generation in the wild, allowing for comparisons between wild fish with known differences in recent hatchery ancestry. Finally, one of the studies (Wenatchee River) also examined how several environmental cofactors contributed to variation in RRS, providing more detailed insight into the role of environmental effects on hatchery salmon RRS. Below, we discuss the analyses presented in each of these studies.

### Genetic effects

**Figure 4:**
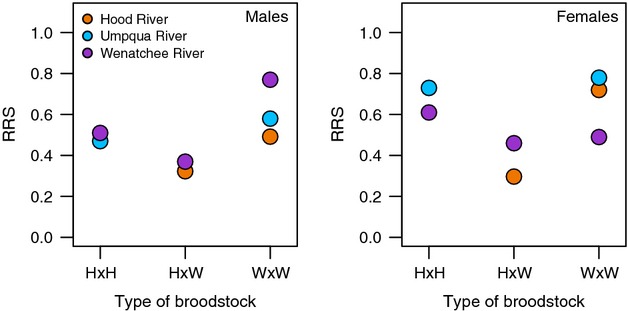
Reproductive success of hatchery fish spawning in the wild relative to wild fish (RRS) calculated for each type of hatchery fish (i.e., those created using wild, hatchery, or both types of broodstock). Three studies conducted different types of broodstock crosses in the hatchery. In the Hood River, two types of broodstock crosses were performed (H × W and W × W; where H equals hatchery and W equals wild). In the Umpqua River, two different types of broodstock crosses were performed (H × H and W × W). In the Wenatchee River, all three crosses were performed. In all cases, only fish from the same run years were compared. In the Hood River, fish with greater amounts of hatchery ancestry had significantly lower reproductive success relative to wild fish (RRS) than those produced by two wild parents, suggesting a genetic effect. In the Umpqua River, there may be a slight trend, but it was not significant, and no trend is present for the Wenatchee River. Note that all RRS estimates for first-generation hatchery fish (W × W) were less than one. RRS, relative reproductive success.

For 5 years in the Hood River, there were two types of crosses performed in the hatchery. The first cross type simply used two wild fish as broodstock (RRS values reported in Figs 1 and 2 are only for hatchery fish created using two wild parents). The second cross type used one wild and one first-generation hatchery fish. When the offspring from both types of crosses spawned in the wild, those created using one hatchery and one wild broodstock parent produced only 55–60% of the number of offspring produced by hatchery fish with two wild parents (Fig. 4; Araki et al. 2007b). Because the only difference between the fish spawning in the wild was the hatchery background of one of their broodstock parents (all hatchery fish were reared together in a common environment), this result suggests a genetic effect.

The Wenatchee Chinook and Umpqua coho studies (Thériault et al. 2011; Ford et al. 2012) followed a similar design to the Hood River steelhead study and compared hatchery fish with different degrees of hatchery ancestry. Unlike the Hood River case, neither study found significant differences in RRS between the different types of hatchery fish spawning in the wild, providing no evidence that reduced RRS was due to genetic effects in these studies (Fig. 4).

Further evidence for genetic effects comes from examining the fitness of wild-born, adult fish as a function of the ancestry of their parents. Araki et al. (2009) is the only study to date that has examined the reproductive success of the wild-born F2 fish in the wild by assigning F3 fish back to their F2 parents (i.e., extending the figure in Box 1 by an additional generation). The F2 wild fish with two F1 hatchery fish as parents had only 37% the fitness of the F2 wild fish that had two wild fish as parents. By looking at the reproductive success of the F2 generation, this study showed that there are substantial ‘carryover’ effects associated with having hatchery parents. This result suggests a genetic effect because all F2 fish spent their entire lives in the wild and illustrates that the fitness reduction created by the hatchery can be passed on to the first wild-born generation.

Additional evidence for genetic effects is found in studies that observed a trade-off in fitness in captivity versus fitness in the wild. Christie et al. (2012a) showed that F1 hatchery fish perform better as broodstock than do wild fish, producing almost twice as many returning hatchery offspring. Also, when comparing among the wild broodstock, those that performed the best in captivity produced fish that performed the worst in the wild. Such trade-offs are consistent with genetic adaptation to the captive environment with a concomitant loss of adaptation to the wild. Using a similar experimental design, Ford et al. (2012) also found a trade-off in reproductive success for the Chinook salmon from the Wenatchee River (case study 1). However, unlike in the Hood River, the trade-off in reproductive success was only found when considering the reproductive success of F1 males in the wild, but was not observed when considering F1 females. In other words, broodstock pairs that performed well in captivity (i.e., had high reproductive success) produced large numbers of F1 male offspring that performed poorly in the wild. This study also provided insight into the mechanism creating the trade-off. The broodstock pairs that produced large numbers of F1 males tended to produce F1 males that returned to spawn as younger and smaller-sized individuals than the F1 males produced by the ‘less successful’ broodstock. The phenomenon of hatchery males returning at younger ages than wild males and therefore being less successful was also seen in the Malbaie River (case study 4; Milot et al. 2013) and has been observed in a variety of other salmon hatchery programs (Hankin et al. 2009; Larsen et al. 2013).

The results reviewed above suggest that reduced reproductive success can accumulate over two or more generations and be passed from parent to offspring. However, none of these studies were designed to thoroughly investigate the genetic architecture of variability in reproductive success. Furthermore, the ‘carryover’ effects of low RRS among generations are consistent with a variety of genetic or epigenetic mechanisms (reviewed by Araki et al. 2008). Additional studies or analyses that more directly explore the genetic or epigenetic mechanisms associated with variance in reproductive success will be important for understanding the evolution of reproductive success over the course of several generations of hatchery supplementation.

### Environmental effects

For Chinook salmon in the Wenatchee River (case study 1), the distribution of spawning location differed between hatchery fish and wild fish and was negatively correlated with reproductive success. F1 hatchery fish tended to spawn lower in the river, and this spawning location was associated with lower reproductive success. Spawning location is determined by a variety of factors, including parental spawning location and the local environment (e.g., Dittman et al. 2010). In the case of Wenatchee River hatchery spring Chinook salmon, spawning location is likely to be largely determined by where the hatchery fish are released as juveniles, which is an environmental factor. At least a portion of the low RRS of Wenatchee River hatchery Chinook salmon is therefore due to environmental factors (Williamson et al. 2010).

## Fitness loss and the salmon life cycle

Results from the case studies highlighted in this article also point to the stage in the life cycle that the fitness-related effects are occurring. It is useful to know when the fitness loss is occurring to predict the extent to which natural selection can purge the population of maladapted individuals (Baskett and Waples 2013). In both the Umpqua and Malbaie rivers, the F1 hatchery fish that were released as fry and survived to return had a higher overall RRS (relative to wild fish) than those that were released as smolts (0.71 for fry vs 0.42 for smolts, Figure S3). Furthermore, for Atlantic salmon, the fish released as fry tended to spend more time at sea than those released as smolts, resulting in fish that were larger and more similar in size to the wild fish. These effects suggest that an extended stay in a hatchery can exacerbate the fitness-related effects associated with hatcheries. However, the fact that the fry still did not have reproductive success equal to that of wild fish suggests that the genetic or environmental effects causing hatchery fish to have low fitness can occur early in the life cycle (e.g., during embryonic development or perhaps owing to relaxed selection on mate choice in their parents). Two studies measured RRS by assigning F2 individuals collected as both juveniles and adults (Berntson et al. 2011; Ford et al. 2013). In both cases, the RRS values calculated by juveniles and adults were similar to one another, which suggests that the reductions in reproductive success in these systems occurred during spawning or early juvenile rearing (as opposed to offspring survival after migration out to the ocean; e.g., Kostow et al. 2003; Reisenbichler et al. 2004).

## Additional studies

In this article, we have focused on studies that directly estimated RRS in the wild from hatchery programs that used local broodstock. However, there are several additional studies that come close to meeting these criteria. For example, at Minter Creek, Washington, the fitness of coho salmon from a long-term supplementation program was evaluated for 2 years (Ford et al. 2006). In this river, there was no barrier to returning hatchery fish, and for several decades prior to the initiation of the study, the ratio of returning hatchery to wild fish may have exceeded 30:1. The authors report a mean RRS (hatchery relative to wild) of 1.01 for males and 0.74 for females, but with neither value significantly different from 1. However, due to the historically high fraction of hatchery fish on the spawning grounds, the authors suggest that there may not have been any detectable differences in fitness because there were no genetically wild fish remaining in the population.

Several studies have also estimated the reproductive success of fish that were introduced into novel environments. For example, Berejikian et al. (2009) examined the fitness of F1 chum salmon (*Oncorhynchus keta*), after the returning adults were transferred to a spawning channel. Fitness was measured by counting fry that were collected in the same channel after using genetic parentage analysis to assign fry to the spawning adults. The overall RRS values equaled 0.83, but the confidence intervals reported in the study ranged from 0.4 to 1.4, indicating that sample sizes may have been insufficient to detect a difference in fitness between the groups. After hatching, chum salmon migrate almost immediately out to sea such that if there is reduced fitness in this system, it would likely represent either relaxed selection in the hatchery (e.g., lack of mate choice) or maternal effects. Another spawning channel study (Schroder et al. 2008) examined the reproductive success of Chinook salmon and observed that the offspring of wild fish had 5.6% greater survival to the fry stage when compared to first-generation hatchery fish (*P* < 0.04). Lastly, a study in which Chinook salmon were allowed to recolonize newly accessible habitat estimated that the RRS equaled 0.83 and 1.48 for males and females, respectively (Anderson et al. 2013). These results are consistent with the two Chinook salmon case studies highlighted above that suggest that males can have lower RRS than females (Fig. 1A, E). While inferences to RRS in natural populations may be more difficult to make with these studies, experimental systems will likely be important for identifying the mechanisms responsible for differences in fitness between hatchery and wild fish.

## Future directions for RRS studies

We provide several recommendations for future studies that examine the reproductive success of wild and early-generation hatchery fish. First, we suggest that new studies target rivers and streams with moderate-to-large population sizes. This may not always be practical, but in the Pacific Northwest, at least, there are a number of streams and rivers supplemented with hatchery fish that have sufficient population sizes to detect even modest effect sizes. Because population size data are often available prior to the initiation of a study, *a priori* power analyses are recommended. Second, we recommend that data be collected from as many F1 run years as possible. Calculating fitness values from multiple years will allow for examination of the between-year variation, reduce the confidence intervals associated with multiyear estimates, and allow for more accurate population-wide estimates of RRS. Lastly, concurrent measurements of environmental variables either may allow for additional explanations of reduced reproductive success or could be used as covariates in statistical models to increase the signal-to-noise ratio (i.e., control for unexplained variation).

In this present analysis, the between-study variation in RRS is not explained by study species. We suspected that steelhead would be more susceptible to reductions in fitness than other species because they spend two or more years in freshwater in the wild, but only 1 year in freshwater in the hatchery before migrating out to sea. However, if we calculate the mean RRS with and without the two steelhead studies (Little Sheep Creek and Hood River), the weighted across-study geometric mean RRS remains essentially unchanged (0.534 vs 0.538). Future RRS studies should include additional species, particularly those with substantially different life-history strategies (e.g., pink salmon, marine fishes), to further determine the generality of the results presented here. We also recommend that future projects evaluate the mechanism responsible for any fitness reduction. Only three of the six case studies highlighted in this article could test for genetic effects (Araki et al. 2007b; Thériault et al. 2011; Ford et al. 2012), and only one study tested for ‘carryover’ effects of hatchery rearing on the reproductive success of their wild-born descendants (Araki et al. 2009). As such, we encourage the development of study designs that will allow for disentangling genetic from environmental effects, particularly those that will allow for the detection of carryover effects (see Box 1). It will also be important to go beyond simply documenting the accumulation of effects across generations and conduct studies that will provide more direct information on the genetic or epigenetic basis responsible for the differences between hatchery and wild salmon.

## Conclusion

Our analyses clearly show that even hatcheries using local- and predominantly wild-origin broodstocks create fish with lower reproductive success than their wild-born counterparts. The point estimates for RRS values across studies were consistently less than one (i.e., 46 of 51 point estimates (90%) were less than one). The reduced fitness of hatchery fish was consistently documented despite differences in geographic location, study species, hatchery practices, and analytical approaches. Therefore, the reduced RRS of early-generation hatchery fish is likely a general phenomenon. It is now vitally important to determine *why* hatchery fish have lower reproductive success in the wild. Answers to that question will pave the way for fisheries management either to implement pragmatic changes to hatchery practices or to re-evaluate the goals of hatchery programs.

## Data archiving statement

Data included in this study were previously published (see literature cited below).

## Supporting Information

**Data S1.** Supporting methods. **Figure S1.** We chose the negative binomial for simulations because it closely mimics the actual distribution of reproductive success values for salmon breeding in the wild. **Figure S2.** The maximum likelihood method of Kalinowski and Taper (2005) was used to calculate 95% confidence intervals for both the single-year and multi-year estimates from each case study. **Figure S3.** Comparison of the relative reproductive success values of F1 hatchery fish that were released as fry or released as smolts. **Figure S4.** An example of the bias that can occur in RRS estimates if fish that produce zero offspring are omitted from calculations. **Table S1.** Example of why it is more appropriate to use the geometric mean rather than the arithmetic mean when averaging RRS estimates across years. **Table S2.** RRS and maximum likelihood confidence intervals calculated for each point estimate included in this study.
