# Supplementary material for: On the reproductive success of early-generation hatchery fish in the wild
Source: Evol Appl. 2014 Jul 9;7(8):883–96. doi: 10.1111/eva.12183 (PMC4211718; doi:10.1111/eva.12183)
Supplement: Supplementary file 1 [file eva0007-0883-sd1.docx]

**Supporting Methods:**

**Sampling, hatchery, and life-history information for each case study:**

*Case 1: Wenatchee River, Chinook salmon:*

The first case study examines the reproductive success of Chinook salmon (*Oncorhynchus tshawytscha*) from the Wenatchee River in Washington State (Williamson *et al.* 2010). The spring-run Chinook from this population have been supplemented with hatchery fish since 1989. In more recent years, this program has produced hatchery-origin fish that provide more than 50% of the individuals on the spawning grounds. In this “stream-type” population, the juvenile salmon migrate to sea after one year in freshwater (Healey 1991). Almost all females return to spawn after an additional three to four years at sea (Age 4-5), as do many males. However, a portion of the male fish adopt an alternative life-history strategy and mature at 1-2 years of age without migrating to sea, and still others spend only a single year at sea and return to breed at three years of age. In this supplementation program, broodstock were taken only from the local population and were comprised of a mixture between wild-origin and first-generation hatchery fish. Between 2004 and 2006, samples of essentially all returning F1 hatchery and wild-born fish were obtained as fish traversed the Tumwater dam (Williamson *et al.* 2010; Ford *et al.* 2013). Males that never went out to sea (or at least those that did not pass below the dam) could not be sampled. Due primarily to differences in annual survival rates between the hatchery and wild components of the population and a tendency for hatchery males to mature earlier than wild males, most F1 hatchery males sampled in 2004 were 2-3 years old, whereas most of the wild males were 4 years old. Because there were a disproportionate number of 2 year-old males that returned in 2004, the sex ratio was highly biased towards males for that particular run-year.

*Case 2: Umpqua River, coho salmon:*

Coho salmon from this population typically spend one year in freshwater before going out to sea for two additional years and returning as reproductively mature adults. A portion of coho males (“jacks”) spend only a single year in the ocean, before returning as smaller males that typically attempt to “sneak” matings. This river system had a prior history of stocking coho from an integrated hatchery program (Thériault *et al.* 2010) that only used local fish as broodstock (~ 30% of broodstock were natural origin). From 2001 through 2003, the Oregon Department of Fish and Wildlife created 100 Hatchery x Hatchery crosses and 100 Wild x Wild crosses in each year. The offspring of all the crosses were split equally between two different rearing environments. The first environment was a typical hatchery setting, in which juvenile coho were reared for a single year before release. After release, the hatchery fish migrated immediately out to sea. The second environment consisted of “hatch boxes” that were placed at various points throughout the river network. Hatch boxes are structural devices placed in a river to protect fertilized salmon eggs until they hatch, at which point the fry are released into the river to associate with other wild-origin fry. The hatch boxes in this study received eyed-eggs and the fry were mixed upon emergence and randomly released at nine sites. All unfed fry and hatchery smolts were released upstream of the Nonpareil dam, which was a barrier to all migrating fishes. Every F1 fish that returned as an adult was sampled as they were passed over the dam. These F1 fish next spawned in the wild and their lifetime reproductive success was evaluated when their adult offspring returned 2-3 years later. This study design allowed for the fitness of three run-years of F1 fish to be evaluated. Furthermore, fitness differences were separated by the sex of the fish, and males were further classified as jacks or three-year-old fish.

*Case 3: Hood River, steelhead:*

The relative fitness of winter-run steelhead (anadromous *Oncorhynchus mykiss*) has been extensively studied in the Hood River, Oregon. Before 1991, a traditional hatchery program was operated that used out-of-basin Big Creek stock (Kostow 2004; Araki *et al.* 2007a). In line with other traditional hatchery programs, the fitness of these out-of-basin fish was reduced by 90% when compared to the wild-born fish (Araki *et al.* 2007a). In 1991, a supplementation program was initiated for the winter-run fish, which consisted of using wild-origin fish as the broodstock. Until its removal in 2010, the Powerdale Dam, located at the mouth of the Hood River, was a complete barrier to all migrating fish. Oregon Department of Fish and Wildlife staff were able to catalog, measure, and obtain fin samples from every adult fish passed over the dam. Thus, this 19 year data set facilitated long-term comparisons of the reproductive success between first-generation hatchery fish from the supplementation program and wild-born steelhead. Steelhead are the anadromous form of *Oncorhynchus mykiss*, and grandparentage analysis revealed that wild-origin resident trout contribute substantially to the production of anadromous steelhead in this population (Christie *et al.* 2011).

*Case 4: Malbaie River, Atlantic salmon:*

This population has been supplemented with first-generation hatchery fish produced by local broodstock since 1992. A dam located near the mouth of the river acts as a barrier to all migrating fishes. As is typical with Atlantic salmon populations, early-maturing resident parr, which were unable to be sampled, may have contributed to subsequent generations of both resident and migrating fish. No first-generation hatchery fish were intentionally used as broodstock. However, there was a possibility that a small portion of broodstock were hatchery-origin fish, as both unmarked fry and smolts were released by the hatchery into the wild. The F1 adults were identified as wild or hatchery origin based on scale aging (as opposed to adipose fin clips) and also via parentage assignment to broodstock parents, though not all broodstock parents could be successfully genotyped. Thus, it is possible that a small portion of F1 fish released as fry were later classified as returning wild-origin fish, but were in fact hatchery-born fish. However, if an F1 hatchery fish was incorrectly assigned as a wild-born fish and if F1 hatchery fish had lower reproductive success than wild-born fish, then the incorrect allocation would only reduce the power to detect a difference in fitness between wild and F1 hatchery fish (i.e., incorrectly allocated F1 fish would reduce the average estimated fitness of the wild fish, reducing the ability to detect a difference). During the summers of 2002-2004, wild-born fry (representing the F2 offspring) were sampled by electro-fishing.

*Case 5: Little Sheep Creek, steelhead:*

The next study examined the lifetime reproductive success in steelhead (*Oncorhynchus mykiss*), from Little Sheep Creek in Oregon (Berntson *et al.* 2011). In 1982, a supportive breeding program was initiated and wild-origin fish were used as broodstock. An effective weir was used to sample all returning F1 hatchery and wild-born adults. A combination of both juvenile and adult F2 fish was used to estimate the reproductive success of F1 fish. Because both hatchery and wild-origin fish were used as broodstock, it is possible that some broodstock had greater proportions of hatchery genes than broodstock from the other case studies. Nevertheless, this study is worth further examination because many supplementation programs may have to rely on F1 hatchery fish as part of their broodstock owing to small population sizes. In this particular case, the population is far inland and has a relatively small population size, such that the number of returning wild-origin fish was often too small to constitute a broodstock collection of sufficient size.

*Case 6: Johnson Creek, Chinook salmon:*

The last case study we included compares the relative reproductive success of Chinook salmon (*Oncorhynchus tshawytscha*) from Johnson Creek, Idaho (Hess *et al.* 2012). A supplementation program was initiated in 1998 and, unlike the previous 5 studies, this river was never used as a release site for prior hatchery programs. Genetic samples were collected from 1998 through 2010 which allowed for adult-to-adult fitness estimates to be made for four run-years. Most hatchery-origin fish, particularly males, returned a year earlier than their wild-born counterparts. Fish were mainly collected at a weir that occurs below 94% of available spawning habitat, though some fish were also collected with field surveys.

**Literature Cited:**

Araki, H., W.R. Ardren, E. Olsen, B. Cooper, and M.S. Blouin. 2007a. Reproductive success of captive-bred steelhead trout in the wild: Evaluation of three hatchery programs in the Hood River. *Conservation Biology* **21**: 181–190.

Araki, H., B. Cooper, and M.S. Blouin. 2007b. Genetic Effects of Captive Breeding Cause a Rapid, Cumulative Fitness Decline in the Wild. *Science* **318**: 100–103.

Berntson, E. A., R.W. Carmichael, M. W. Flesher, E. J. Ward, and P. Moran. 2011. Diminished Reproductive Success of Steelhead from a Hatchery Supplementation Program (Little Sheep Creek, Imnaha Basin, Oregon). *Transactions of the American Fisheries Society* **140**: 685–698.

Christie, M. R., M. L. Marine, and M. S. Blouin. 2011. Who are the missing parents? Grandparentage analysis identifies multiple sources of gene flow. *Molecular Ecology* **20**: 1263-1276.

Ford M.J., S. Howard, A.R. Murdoch, M.S. Hughes. 2013. Monitoring the reproductive success of naturally spawning hatchery and natural spring Chinook salmon in the Wenatchee River. Report to the Bonneville Power Administration on project 2003-039-00. May 2013. Available from <https://pisces.bpa.gov/release/documents/documentviewer.aspx?doc=P132194>.

Healey M. 1991. Life history of Chinook salmon (*Oncorhynchus tshawytscha*). *Pacific salmon life histories*, 313-393.

Hess, M. A., C. D. Rabe, J. L. Vogel, J. J. Stephenson, D. D. Nelson, and S. R. Narum. 2012. Supportive Breeding Boosts Natural Population Abundance with Minimal Negative Impacts on Fitness of a Wild Population of Chinook Salmon. *Molecular Ecology* **21**: 5236–5250.

Kostow, K. E. 2004. Differences in Juvenile Phenotypes and Survival Between Hatchery Stocks and a Natural Population Provide Evidence for Modified Selection Due to Captive Breeding. *Canadian Journal of Fisheries and Aquatic Sciences* **61**: 577–589.

Milot, E., C. Perrier, L. Papillon, J. J. Dodson, and L. Bernatchez. 2013. Reduced Fitness of Atlantic Salmon Released in the Wild after One Generation of Captive Breeding. *Evolutionary Applications* **6**: 472–485.

Thériault, V., G. R. Moyer, and M. A. Banks. 2010. Survival and Life History Characteristics Among Wild and Hatchery Coho Salmon (*Oncorhynchus Kisutch*) Returns: How Do Unfed Fry Differ from Smolt Releases? *Canadian Journal of Fisheries and Aquatic Sciences* **67**:486–497.

Thériault, V., G. R. Moyer, L. S. Jackson, M. S. Blouin, and M. A. Banks. 2011. Reduced Reproductive Success of Hatchery Coho Salmon in the Wild: Insights into Most Likely Mechanisms. *Molecular Ecology*  **20**: 1860–1869.

Williamson, K. S., A. R. Murdoch, T. N. Pearsons, E. J. Ward, and M. J. Ford. 2010. Factors Influencing the Relative Fitness of Hatchery and Wild Spring Chinook Salmon (*Oncorhynchus tshawytscha*) in the Wenatchee River, Washington, USA. *Canadian Journal of Fisheries and Aquatic Sciences* **67**:1840–1851.

**Figure S1:** We chose the negative binomial for simulations because it closely mimics the actual distribution of reproductive success values for salmon breeding in the wild. A.) An example of a distribution created with the negative binomial distribution. B.) An observed distribution of family sizes from wild winter-run steelhead from the Hood River, Oregon. Notice that 0 and 1 were the most commonly observed values in both plots.

**
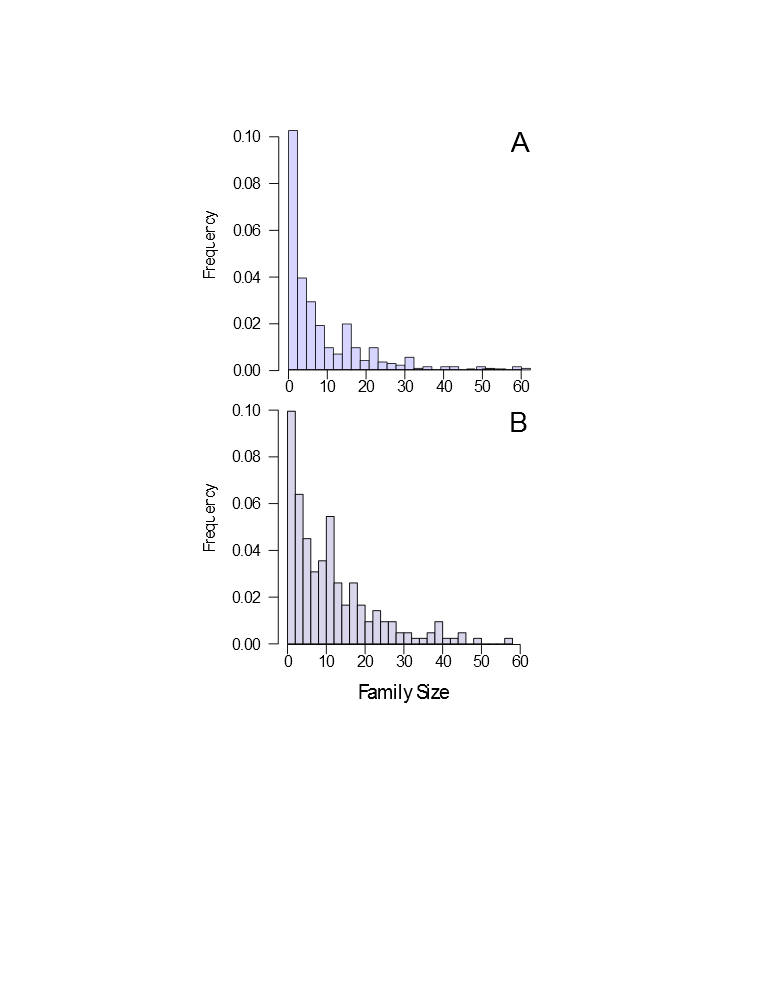
**

Frequency

**Figure S2:** The maximum likelihood method of Kalinowski and Taper (2005) was used to calculate 95% confidence intervals for both the single-year and multi-year estimates from each case study. The range of the 95% confidence interval (i.e., the difference between the upper and lower confidence limits) for multi-year estimates (blue points) was substantially smaller than for single-year estimates (green points). Males and females are plotted sequentially for each study. Case studies are as follows: 1 Wenatchee River; 2 Umpqua River; 3 Hood River; 4 Malbaie River; 5 Little Sheep Creek; 6 Johnson Creek (see Table 1 for details).


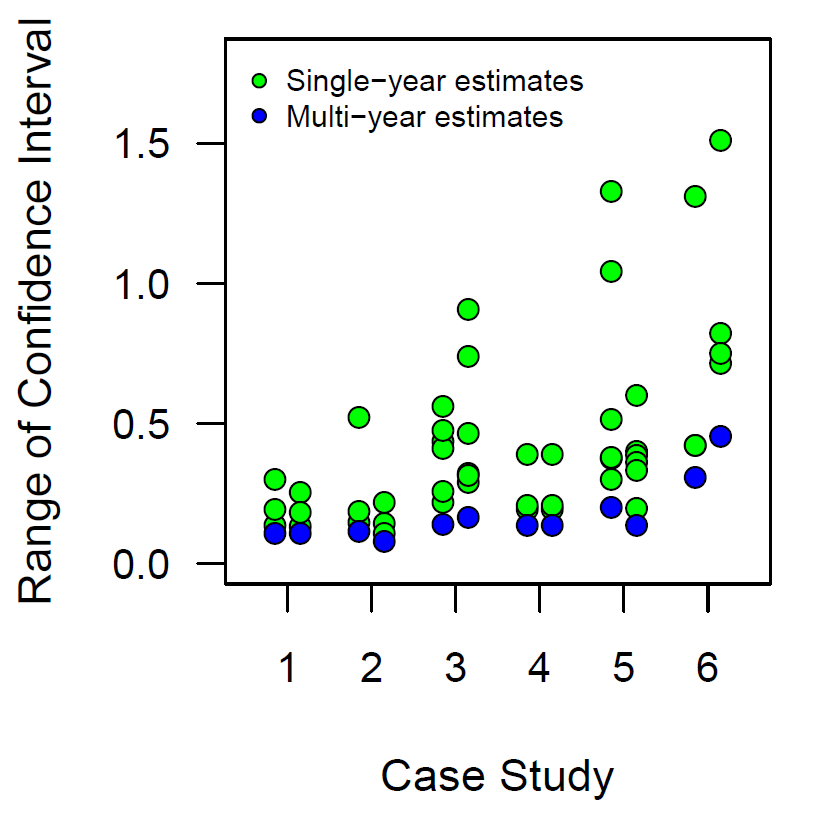


**Figure S3:** Comparison of the relative reproductive success values of F1 hatchery fish that were released as fry or released as smolts. Purple circles represent data from coho salmon. In 5 of 6 cases, the fish released as fry had higher reproductive success than the fish released as smolts. Green circles represent data from Atlantic salmon, and in one year there was a substantial difference. Arrows depict the mean RRS for fry and smolts, respectively. Because all of the RRS values are below 1 (including for fish released as fry), these data suggest that the negative effects associated with hatchery culture may occur early in the life cycle. The difference between fish released as fry *versus* as smolts suggests that negative effects continue to accrue as fish spend longer periods of time in the hatchery.


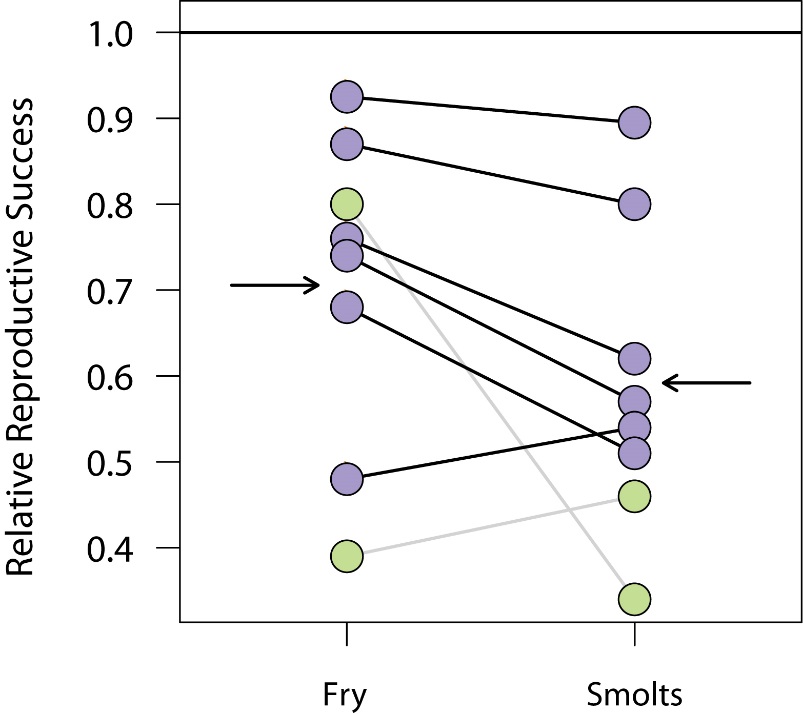


**Figure S4:** An example of the bias that can occur in RRS estimates if fish that produce zero offspring are omitted from calculations. Here, we used a negative binomial distribution and simulated the ‘hatchery fish’ to have a lower fitness, and thus a greater proportion of individuals that left no surviving offspring (‘zero’). For each RRS estimate the same distributions were used except that individuals that produced zero offspring were either included (‘RRS with zero’) or excluded (‘RRS without zero’) from the calculations. The procedure was repeated 1000 times for each point and the mean was recorded. The X-axis shows the true RRS (fitness of hatchery fish relative to that of wild fish), while the Y-axis shows the RRS one would estimate after deleting the zero-class fish. If there were no bias, then the points would fall along the 1:1 line. Here we see that as true RRS decreases (i.e. as the difference in fitness between hatchery and wild fish becomes larger) the upward bias in favor of hatchery fish is exacerbated. This is because, as the true RRS decreases, the proportion of hatchery fish that leave zero offspring increases relative to the proportion of wild fish that leave zero offspring. The problem with pair data (i.e. RRS analyses that examine the fitness of particular *cross types* of F1 fish) is that it is not possible to identify pairs that left 0 surviving offspring. If one particular cross type has more pairs that leave zero surviving offspring, then the RSS estimates would be biased as illustrated in this example.

**
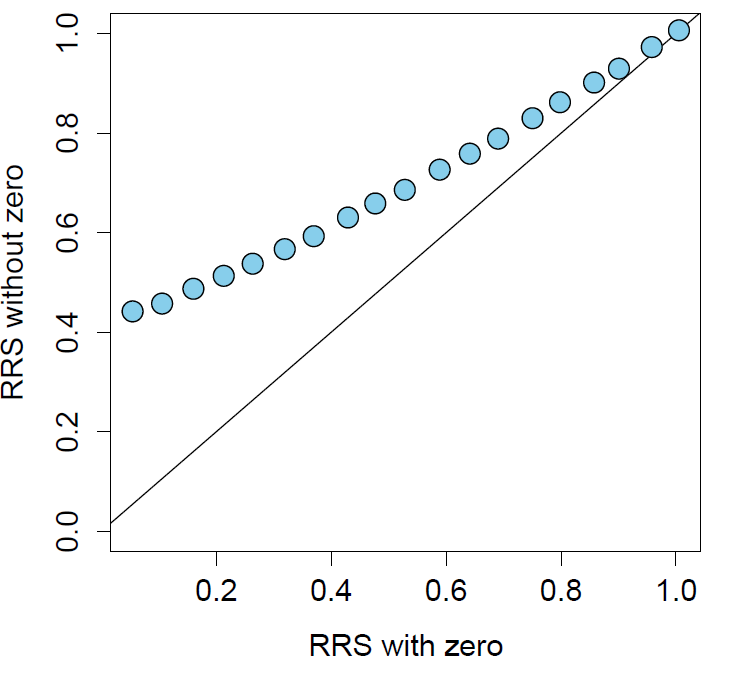
**

**Table S1:** Example of why it is more appropriate to use the geometric mean rather than the arithmetic mean when averaging RRS estimates across years. Note that in RRS studies, the fitness of hatchery fish is arbitrarily placed in the numerator. Using estimates of fitness from 3 years as an example, we can see that RRS would be calculated as 1.167 using the arithmetic mean. However, if RRS were calculated as the fitness of wild/hatchery fish, then we would still calculate RRS as 1.167. Thus, depending on what was placed in the numerator the estimate of RRS would be biased towards wild or hatchery fish. Notice that this problem is avoided entirely by calculating the geometric mean.

| \| **Group** \| **Year1** \| **Year2** \| **Year3** \| **Arithmetic Mean** \| **Geometric Mean** \| \| --- \| --- \| --- \| --- \| --- \| --- \| \| Hatchery fitness \| 1 \| 2 \| 1 \|  \|  \| \| Wild fitness \| 2 \| 1 \| 1 \|  \|  \| \| H/W \| 0.5 \| 2 \| 1 \| 1.167 \| 1 \| \| W/H \| 2 \| 0.5 \| 1 \| 1.167 \| 1 \| |  |  |  |  |
| --- | --- | --- | --- | --- | --- | --- | --- | --- | --- | --- | --- | --- | --- | --- | --- | --- | --- | --- | --- | --- | --- | --- | --- | --- | --- | --- | --- | --- | --- | --- | --- | --- | --- | --- |

**Table S2:** RRS and maximum likelihood confidence intervals calculated for each point estimate included in this study. We also include the sample sizes for F1 wild (Nwild) and F1 hatchery (Nhat) used in each study. The lower and upper CI’s represent the lower and upper 95% confidence intervals, respectively. RRS.ml is the maximum likelihood estimate of RRS.

| **Case Study** | **F1_runyear** | **Species** | **Sex** | **Nwild** | **Nhat** | **RRS** | **RRS_ml** | **Lower CI** | **Upper CI** |
| --- | --- | --- | --- | --- | --- | --- | --- | --- | --- |
| 1 | 2004 | Chinook | M | 392 | 93 | 0.411 | 0.411 | 0.281 | 0.581 |
| 1 | 2005 | Chinook | M | 226 | 1118 | 0.392 | 0.392 | 0.330 | 0.469 |
| 1 | 2006 | Chinook | M | 184 | 372 | 0.522 | 0.522 | 0.434 | 0.627 |
| 1 | Combined | Chinook | M |  |  |  | 0.446 | 0.395 | 0.503 |
| 1 | 2004 | Chinook | F | 359 | 245 | 0.677 | 0.677 | 0.559 | 0.816 |
| 1 | 2005 | Chinook | F | 238 | 1676 | 0.388 | 0.388 | 0.327 | 0.462 |
| 1 | 2006 | Chinook | F | 248 | 716 | 0.603 | 0.603 | 0.519 | 0.702 |
| 1 | Combined | Chinook | F |  |  |  | 0.544 | 0.492 | 0.602 |
| 2 | 2004 | Coho | F | 358 | 135 | 0.910 | 0.910 | 0.680 | 1.202 |
| 2 | 2005 | Coho | F | 352 | 361 | 0.620 | 0.620 | 0.549 | 0.699 |
| 2 | 2006 | Coho | F | 218 | 295 | 0.800 | 0.800 | 0.711 | 0.900 |
| 2 | Combined | Coho | F |  |  | 0.720 | 0.719 | 0.664 | 0.780 |
| 2 | 2004 | Coho | M | 384 | 156 | 0.570 | 0.570 | 0.468 | 0.689 |
| 2 | 2005 | Coho | M | 277 | 287 | 0.540 | 0.540 | 0.472 | 0.617 |
| 2 | 2006 | Coho | M | 188 | 267 | 0.510 | 0.510 | 0.459 | 0.567 |
| 2 | Combined | Coho | M |  |  | 0.530 | 0.528 | 0.489 | 0.570 |
| 3 | 1995 | Steelhead | F | 131 | 66 | 0.771 | 0.771 | 0.623 | 0.948 |
| 3 | 1996 | Steelhead | F | 147 | 153 | 0.932 | 0.932 | 0.797 | 1.090 |
| 3 | 1997 | Steelhead | F | 110 | 106 | 1.260 | 1.260 | 1.049 | 1.515 |
| 3 | 1998 | Steelhead | F | 166 | 91 | 0.503 | 0.503 | 0.365 | 0.681 |
| 3 | 1999 | Steelhead | F | 538 | 110 | 0.725 | 0.725 | 0.373 | 1.282 |
| 3 | 2000 | Steelhead | F | 578 | 290 | 0.968 | 0.968 | 0.659 | 1.398 |
| 3 | Combined | Steelhead | F |  |  | 0.910 | 0.906 | 0.826 | 0.993 |
| 3 | 1995 | Steelhead | M | 78 | 92 | 0.673 | 0.673 | 0.571 | 0.793 |
| 3 | 1996 | Steelhead | M | 94 | 95 | 1.050 | 1.050 | 0.854 | 1.291 |
| 3 | 1997 | Steelhead | M | 67 | 53 | 0.846 | 0.846 | 0.607 | 1.170 |
| 3 | 1998 | Steelhead | M | 90 | 95 | 0.492 | 0.492 | 0.378 | 0.636 |
| 3 | 1999 | Steelhead | M | 338 | 111 | 0.377 | 0.377 | 0.212 | 0.623 |
| 3 | 2000 | Steelhead | M | 301 | 279 | 0.741 | 0.741 | 0.538 | 1.014 |
| 3 | Combined | Steelhead | M |  |  | 0.710 | 0.714 | 0.647 | 0.789 |
| 4 | 2002 | Atlantic salmon | Both | 120 | 15 | 0.300 | 0.296 | 0.144 | 0.534 |
| 4 | 2003 | Atlantic salmon | Both | 167 | 86 | 0.450 | 0.446 | 0.356 | 0.552 |
| 4 | 2004 | Atlantic salmon | Both | 206 | 142 | 0.640 | 0.637 | 0.540 | 0.750 |
| 4 | Combined | Atlantic salmon | Both |  |  | 0.540 | 0.538 | 0.473 | 0.610 |
| 5 | 2000 | Steelhead | M | 35 | 49 | 0.513 | 0.513 | 0.313 | 0.830 |
| 5 | 2001 | Steelhead | M | 34 | 331 | 0.728 | 0.728 | 0.405 | 1.449 |
| 5 | 2002 | Steelhead | M | 63 | 453 | 0.229 | 0.229 | 0.127 | 0.427 |
| 5 | 2003 | Steelhead | M | 47 | 163 | 0.737 | 0.737 | 0.353 | 1.681 |
| 5 | 2004 | Steelhead | M | 76 | 392 | 0.414 | 0.414 | 0.271 | 0.647 |
| 5 | 2005 | Steelhead | M | 78 | 159 | 0.275 | 0.275 | 0.139 | 0.521 |
| 5 | Combined | Steelhead | M |  |  | 0.441 | 0.441 | 0.352 | 0.554 |
| 5 | 2000 | Steelhead | F | 23 | 91 | 0.546 | 0.546 | 0.384 | 0.787 |
| 5 | 2001 | Steelhead | F | 66 | 347 | 0.481 | 0.481 | 0.330 | 0.716 |
| 5 | 2002 | Steelhead | F | 129 | 728 | 0.347 | 0.347 | 0.215 | 0.577 |
| 5 | 2003 | Steelhead | F | 46 | 153 | 0.432 | 0.432 | 0.230 | 0.833 |
| 5 | 2004 | Steelhead | F | 60 | 291 | 0.268 | 0.268 | 0.188 | 0.385 |
| 5 | 2005 | Steelhead | F | 98 | 129 | 0.297 | 0.297 | 0.168 | 0.503 |
| 5 | Combined | Steelhead | F |  |  | 0.393 | 0.393 | 0.331 | 0.468 |
| 6 | 2002 | Chinook | M | 155 | 255 | 0.430 | 0.423 | 0.259 | 0.682 |
| 6 | 2003 | Chinook | M | 100 | 29 | 0.910 | 0.895 | 0.420 | 1.731 |
| 6 | 2004 | Chinook | M | 50 | 42 | 0.680 | 0.689 | 0.506 | 0.929 |
| 6 | Combined | Chinook | M |  |  | 0.620 | 0.624 | 0.487 | 0.796 |
| 6 | 2002 | Chinook | F | 42 | 167 | 0.550 | 0.553 | 0.311 | 1.026 |
| 6 | 2003 | Chinook | F | 165 | 87 | 0.840 | 0.853 | 0.525 | 1.349 |
| 6 | 2004 | Chinook | F | 41 | 39 | 1.300 | 1.278 | 0.959 | 1.709 |
| 6 | 2005 | Chinook | F | 4 | 8 | 1.190 | 1.133 | 0.629 | 2.142 |
| 6 | Combined | Chinook | F |  |  | 1.050 | 1.051 | 0.849 | 1.302 |
